# Network pharmacology and molecular simulation study on the multi-target gene regulatory mechanism of *Isatis indigotica* against hepatic inflammation

**DOI:** 10.1371/journal.pone.0355930

**Published:** 2026-08-13

**Authors:** XueRu Li, Yang Wang, HuiJun Cheng, Rong Chen, XiaoLu Chen

**Affiliations:** 1 College of Biological Sciences and Technology, Yili Normal University, Yining, Xinjiang, China; 2 Key Laboratory of Microbial Resources Protection, Development and Utilization, Yili Normal University, Yining, Xinjiang, China; Guangdong Nephrotic Drug Engineering Technology Research Center, Institute of Consun Co. for Chinese Medicine in Kidney Diseases, CHINA

## Abstract

*Isatis indigotica* (Banlangen) is a classic Traditional Chinese Medicine herbal remedy with well-documented antiviral and anti-inflammatory properties. However, the molecular mechanisms underlying its therapeutic effects against hepatitis B virus (HBV)-associated hepatic inflammation remain incompletely understood. This study aimed to systematically elucidate the multi-target regulatory mechanisms of *Isatis indigotica* against HBV-associated hepatic inflammation using network pharmacology and molecular simulation approaches. Bioactive compounds were screened from the TCMSP database (OB ≥ 30%, oral bioavailability; DL ≥ 0.18, drug-likeness). Candidate targets were identified by integrating SwissTargetPrediction with GeneCards/OMIM disease targets. Protein-protein interaction (PPI) network topology analysis identified hub genes. Gene Ontology (GO) and Kyoto Encyclopedia of Genes and Genomes (KEGG) pathway enrichment analyses were performed using Metascape. Molecular docking and 100 ns all-atom molecular dynamics (MD) simulation were conducted to validate compound-target binding. Seventeen bioactive compounds were identified, yielding 1,023 compound targets. Intersecting with 1,676 HBV disease targets produced 142 candidate genes. PPI network analysis identified *AKT1*, *IL6*, *TP53*, and *TNF* as hub genes, significantly enriched in NF-κB, JAK-STAT, and TNF signaling pathways (*P* < 0.01). Molecular docking confirmed favorable binding affinities, with IQ (6-(3-oxoindolin-2-ylidene)indolo[2,1-b]quinazolin-12-one) showing optimal binding to *AKT1* (ΔG = −9.35 kcal/mol). MD simulation verified stable binding over 100 ns. This network pharmacology study suggests that *Isatis indigotica* acts on HBV-associated hepatic inflammation through multi-target synergistic regulation of NF-κB and JAK-STAT signaling pathways, providing mechanistic insights and potential therapeutic targets for HBV management.

## 1. Introduction

Hepatic inflammation serves as a central pathological mechanism in the progression of various liver diseases, with its molecular basis involving abnormal activation of inflammation-related genes and disruption of regulatory networks [1–3]. In chronic hepatitis B, persistent hepatitis B virus (HBV) infection can induce host immune response disorders, driving aberrant expression of key inflammatory regulatory genes such as *AKT1* and *TNF*, thereby establishing a chronic inflammatory microenvironment that significantly increases the risk of hepatic fibrosis, cirrhosis, and hepatocellular carcinoma [4,5]. Although nucleos(t)ide analogs and pegylated interferon can effectively suppress viral replication, their efficacy in reversing established inflammatory damage and immune exhaustion remains limited [6,7]. Therefore, targeted synergistic regulation of multiple inflammatory gene nodes has emerged as a critical strategy for developing novel anti-hepatic inflammation approaches.

*Isatis indigotica* Fort. (Banlangen), a classic antipyretic and detoxifying herbal medicine in Traditional Chinese Medicine (TCM), has been demonstrated by modern research to possess significant antiviral, anti-inflammatory, and immunomodulatory activities [8,9]. However, due to the chemical complexity of *Isatis indigotica*, the traditional “single-component–single-target” research paradigm inadequately explains its synergistic regulatory mechanisms on inflammatory gene networks, thereby constraining deeper mechanistic understanding and clinical translation.

Network pharmacology, by constructing multi-dimensional regulatory networks of “component–target gene–pathway,” aligns with the holistic perspective and systemic regulatory characteristics of TCM, providing a novel strategy for deciphering gene-level mechanisms of complex herbal systems [10]. This integrated strategy has been successfully applied to the study of Food and medicine homology substances. For instance, Liu et al. employed network pharmacology to identify key targets of mulberry leaf extract in metabolic dysfunction-associated fatty liver disease (MAFLD), and then validated their predictions via animal experiments [11]. While several pioneers have mapped the basic network pharmacology landscape of *Isatis indigotica* against HBV, their findings were limited to static network topologies and generic inflammatory hubs [12]. Such topology-driven approaches are intrinsically biased toward highly interconnected nodes, often yielding recurrent generic signaling hubs across disparate disease models rather than context-specific therapeutic targets [13]. To overcome the limitations of static network inference, molecular docking and molecular dynamics (MD) simulations have emerged as gold-standard computational tools for validating ligand–target binding modes and conformational stability under physiological conditions [14]. Integrating network pharmacology with molecular docking and MD simulations constitutes a powerful in silico pipeline that bridges systems-level target prediction with atomic-level structural evidence [15]. The present work uniquely advances this field by combining network pharmacology with molecular docking and MD simulation, which enables validation of binding modes and stability between bioactive components and target gene products at the protein structural level, offering structural biological evidence for network predictions. Although several network pharmacology studies on *Isatis indigotica* have reported similar inflammatory pathways, the present study provides three distinct advances: (1) exclusive application of stringent ‘highest confidence’ (score >0.9) PPI filtering to reduce false positives; (2) 100 ns all-atom MD simulation to validate binding stability, which is rarely performed in previous studies; and (3) identification of IQ as a potential AKT1 stabilizer, a hypothesis not previously proposed.

Based on this rationale, the present study integrated network pharmacology-based target gene prediction, molecular docking validation, and MD simulation to systematically identify core bioactive components and key target genes of *Isatis indigotica* against HBV-associated hepatic inflammation, elucidating its multi-target synergistic anti-inflammatory mechanisms from a gene regulatory network perspective and providing a mechanistic foundation for modern research on *Isatis indigotica* and anti-hepatitis drug development.

## 2. Materials and methods

Ethics statement: This study is a purely computational analysis involving no human participants, animal subjects, or biological samples. All data were retrieved from publicly available databases and published literature. Therefore, no ethics committee approval was required.

### 2.1. Bioactive compound identification and target prediction

Using “*Isatis indigotica*” as the search keyword, chemical constituents were retrieved from the Traditional Chinese Medicine Systems Pharmacology Database and Analysis Platform (TCMSP, https://www.tcmsp-e.com/). Screening thresholds were set at OB (oral bioavailability) ≥30% and DL (drug-likeness) ≥0.18, following established criteria for candidate bioactive compound selection. After removing duplicates, candidate bioactive compounds were obtained. SMILES structural formulas were retrieved from the PubChem database (https://pubchem.ncbi.nlm.nih.gov/) and imported into the SwissTargetPrediction server (http://www.swisstargetprediction.ch/) with the organism set to “*Homo sapiens*.” Targets with prediction probability scores > 0 were selected as potential targets of the respective compounds.

### 2.2. Identification of HBV-associated disease targets

Using “hepatitis B virus” as the search keyword, disease-associated targets were retrieved from the GeneCards database (https://www.genecards.org/) and the OMIM database (https://www.omim.org/). The union of results from both databases was obtained. For GeneCards, targets with relevance scores above the median value were retained to ensure target quality.

### 2.3. Identification of intersection targets

The Venny 2.1.0 online tool (https://bioinfogp.cnb.csic.es/tools/venny/) was used to identify the intersection between *Isatis indigotica* compound targets and HBV disease targets. The resulting intersection targets represent potential therapeutic targets of *Isatis indigotica* in the context of HBV-associated hepatic inflammation.

### 2.4. Construction and analysis of compound–target network

Data on *Isatis indigotica* bioactive compounds and the 142 intersection targets were imported into Cytoscape software (version 3.10.3; https://cytoscape.org/) to construct a visualized compound–target–disease network. Network topology parameters (degree, betweenness centrality, closeness centrality) were calculated using the built-in Network Analyzer plug-in. Core bioactive compounds and key targets were ranked by degree value, with higher values indicating more critical regulatory roles in the network.

### 2.5. Construction and analysis of protein–protein interaction network

Intersection targets were imported into the STRING database (https://cn.string-db.org/) with the species set to “*Homo sapiens*” and the minimum interaction confidence threshold set to “highest confidence” (score > 0.9). Isolated nodes were removed prior to protein–protein interaction (PPI) analysis. The resulting network was imported into Cytoscape 3.10.3. Using the CytoNCA plug-in, nodes were ranked by degree centrality to identify core hub targets.

### 2.6. GO and KEGG pathway enrichment analysis

GO functional enrichment analysis and KEGG pathway enrichment analysis of the 142 intersection targets were performed using the Metascape platform (https://metascape.org/). A significance threshold of *P* < 0.01 was applied to identify significantly enriched biological processes, cellular components, molecular functions, and signaling pathways. Enrichment results were visualized using the online Bioinformatics platform (https://www.bioinformatics.com.cn/).

### 2.7. Molecular docking

Three-dimensional crystal structures of core target proteins were downloaded from the Protein Data Bank (https://www.rcsb.org/) and preprocessed using PyMOL for water molecule removal and co-crystallized ligand extraction. Mol2-format structures of major bioactive compounds were obtained from the TCMSP database. AutoDockTools (version 1.5.6) was used for receptor and ligand preparation, including addition of polar hydrogen atoms and Gasteiger charge calculation. Molecular docking was performed using AutoDock Vina with a grid box encompassing the active site of each target protein. A binding free energy ≤−5.0 kcal/mol was considered indicative of favorable binding activity. Docking poses were visualized using PyMOL and Discovery Studio Visualizer.

### 2.8. Molecular dynamics simulation

The protein–ligand complex with the optimal molecular docking binding affinity was selected for a 100 ns all-atom MD simulation to validate the long-term binding stability between the compound and the target protein. Simulations were performed using the GROMACS software package (version 2021.4). The protein was parameterized using the AMBER99SB-ILDN all-atom force field. Ligand topology parameters were generated using Sobtop (version 1.0 dev5). The system was placed in a dodecahedral periodic boundary condition box with a minimum distance of 1.2 nm between solute atoms and box walls. The system was solvated using the TIP3P explicit water model and neutralized with Na ^+^ /Cl⁻ counter-ions. Energy minimization was performed using the steepest descent algorithm until the maximum force converged below 1000 kJ mol ⁻ ¹ nm ⁻ ¹. The system was subsequently equilibrated under NVT (300 K, 100 ps, leap-frog integrator) and NPT (1 bar, 100 ps, Berendsen pressure coupling) ensembles. Production MD simulation was conducted for 100 ns with an integration time step of 1 fs, and trajectory frames were saved every 10 ps. Long-range electrostatic interactions were treated using the Particle Mesh Ewald method. Trajectory post-processing was performed using gmx trjconv with the -pbc nojump flag to remove periodic boundary condition artifacts. Root-mean-square deviation (RMSD), root-mean-square fluctuation (RMSF), radius of gyration (Rg), hydrogen bond analysis, solvent-accessible surface area (SASA), and potential energy were analyzed using standard GROMACS analysis tools. Free energy landscape (FEL) analysis was performed using the gmx sham script with Rg and RMSD as reaction coordinates. Results were visualized using QtGrace (version 5.0.3).

## 3. Results

### 3.1. Bioactive compound identification and target prediction

Based on the screening criteria of OB ≥ 30% and DL ≥ 0.18, a total of 17 candidate bioactive compounds were identified through TCMSP and PubChem database searches (Table 1). Target information for each bioactive compound was retrieved from the SwissTargetPrediction server. After screening, combining, and removing duplicates, a total of 1,023 potential compound targets were obtained.

**Table 1:** Bioactive compounds of *Isatis indigotica* and their properties (OB ≥ 30%, DL ≥ 0.18).

MOL ID	Chemical compound	OB(%)	DL	Number of targets
MOL001689	acacetin	34.97	0.24	100
MOL002322	isovitexin	31.29	0.72	3
MOL001733	eupatilin	30.23	0.37	100
MOL001734	3-[[(2R,3R,5R,6S)-3,5-dihydroxy-6-(1H-indol-3-yloxy)-4-oxooxan-2-yl]methoxy]-3-oxopropanoic acid	85.87	0.47	22
MOL001735	dinatin (hispidulin)	30.97	0.27	100
MOL001755	24-Ethylcholest-4-en-3-one	36.08	0.76	46
MOL001756	quindoline	33.17	0.22	17
MOL001771	poriferast-5-en-3beta-ol	36.91	0.75	40
MOL001774	ineketone	37.14	0.3	100
MOL001779	sinoacutine	49.11	0.46	78
MOL001781	indigo	38.2	0.26	12
MOL001790	linarin	39.84	0.71	20
MOL001792	DFV	32.76	0.18	100
MOL001803	sinensetin	50.56	0.45	100
MOL001810	IQ (6-(3-oxoindolin-2-ylidene)indolo[2,1-b]quinazolin-12-one)	45.28	0.89	100
MOL000358	beta-sitosterol	36.91	0.75	43
MOL000449	stigmasterol	43.83	0.76	42

### 3.2. HBV-associated disease targets and intersection targets

**Fig 1:**
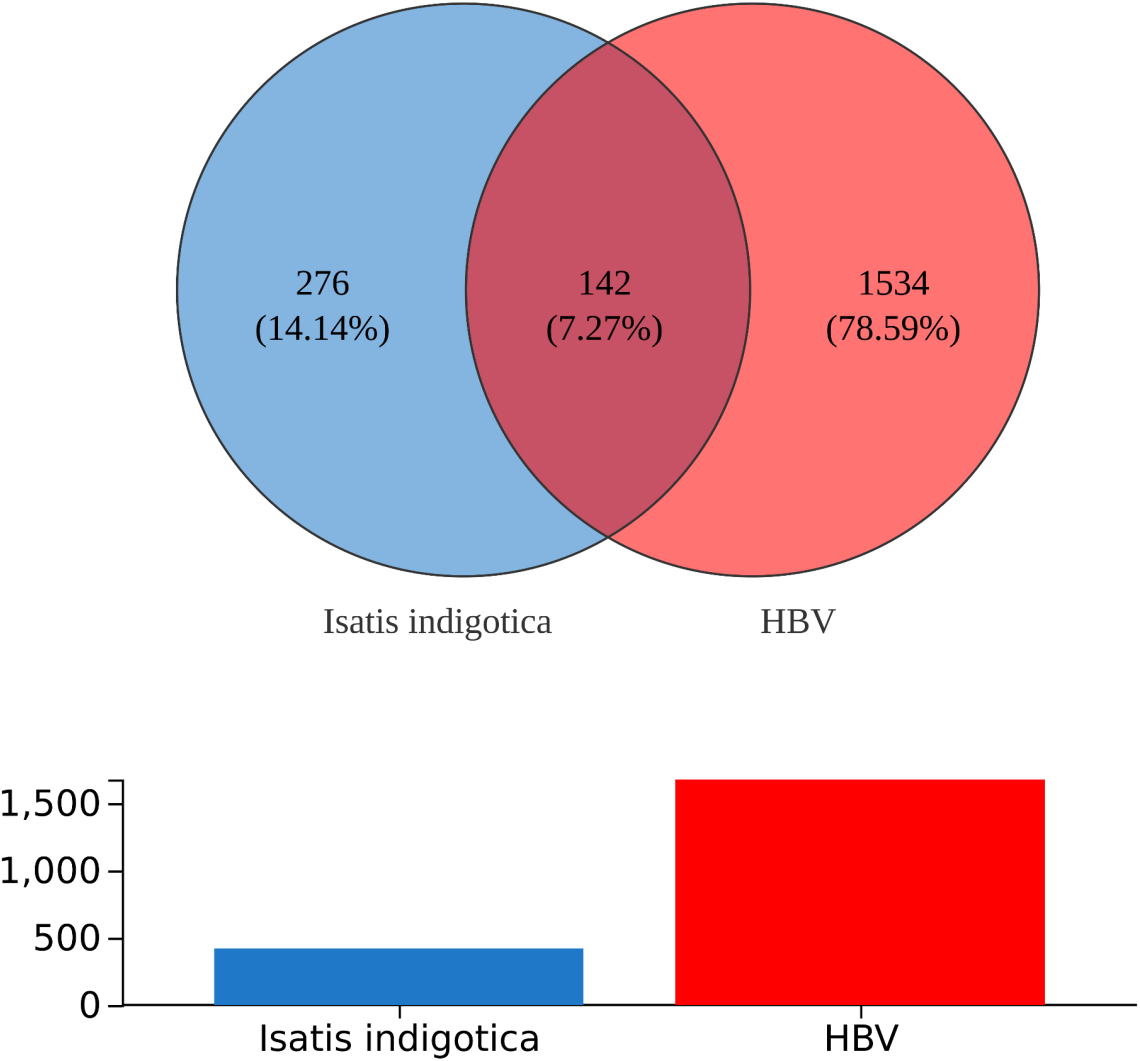
Venn diagram of *Isatis indigotica* compound targets and HBV-associated disease targets. The intersection region contains 142 shared targets representing candidate therapeutic targets for *Isatis indigotica* against HBV-associated hepatic inflammation.

A total of 1,676 HBV-related targets were retrieved and filtered from the GeneCards and OMIM databases. Venn diagram analysis using Venny 2.1.0 identified 142 intersection targets shared between 1,023 *Isatis indigotica* compound targets and 1,676 HBV disease targets (Fig 1; full list in S1 Table).

### 3.3. Compound–target network analysis

**Fig 2:**
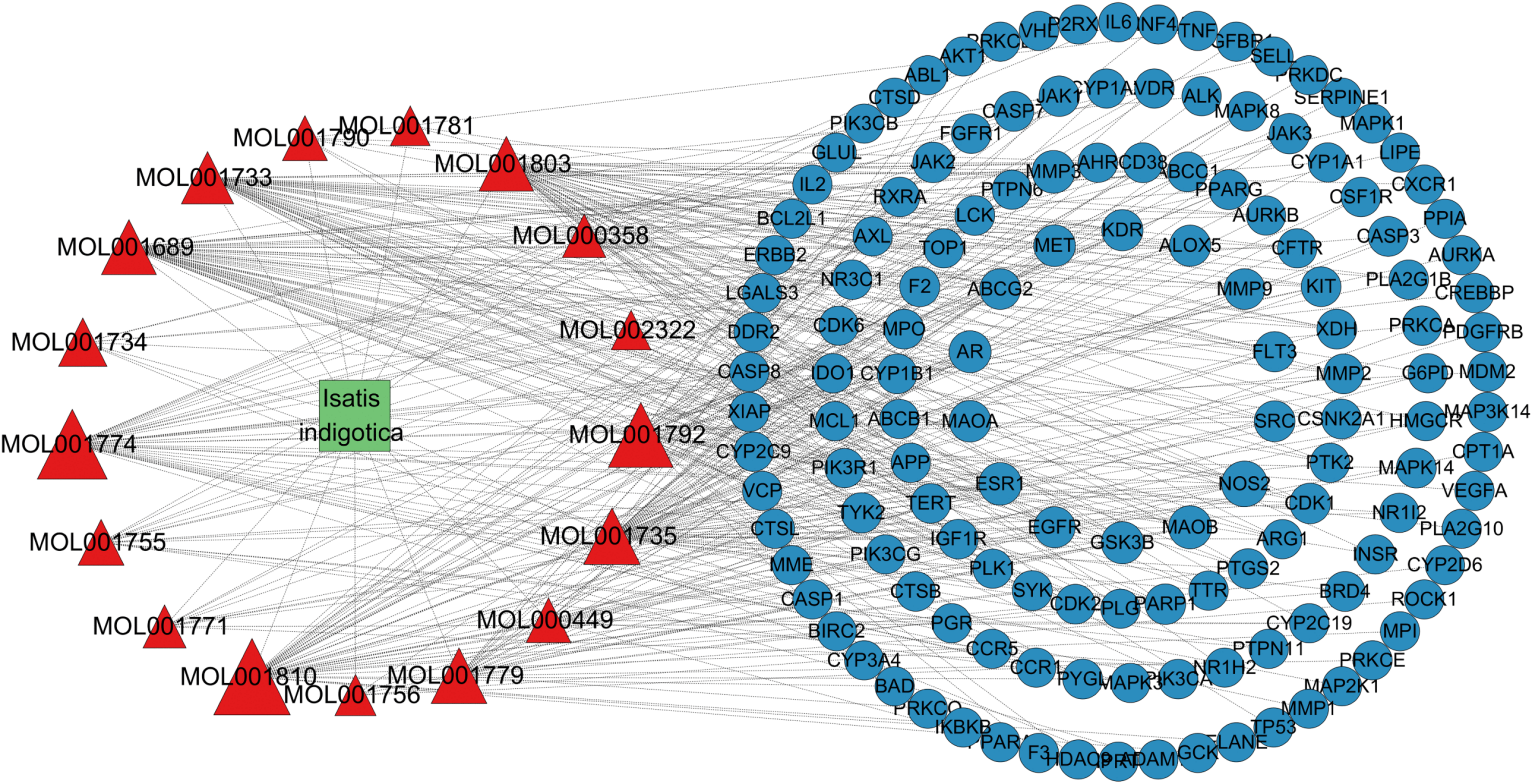
Compound–target network of *Isatis indigotica* against HBV-associated hepatic inflammation. Green nodes represent bioactive compounds; blue nodes represent intersection targets. Node size and color depth are proportional to degree value.

The 17 bioactive compounds of *Isatis indigotica* and their 142 intersection targets with HBV were imported into Cytoscape 3.10.3 to construct a visualized “compound–intersection target–HBV” network. This network contained 159 nodes (17 bioactive compounds and 142 core targets) and 526 interaction relationships (Fig 2). Green nodes represent bioactive compounds of *Isatis indigotica*, and blue nodes represent intersection targets; larger node size and deeper color indicate higher degree values and greater regulatory importance. Network topology analysis revealed that all bioactive compounds exhibit “one-to-many” target interaction characteristics, consistent with the polypharmacological synergistic regulatory mechanisms characteristic of TCM. Based on degree value ranking, acacetin, dinatin, sinensetin, eupatilin, and (IQ)6-(3-oxoindolin-2-ylidene)indolo[2,1-b]quinazolin-12-one were identified as the compounds most likely to exert major regulatory roles in HBV prevention and treatment (top 10 compounds listed in S2 Table). The complete compound-target network data are provided in S3 Table.

### 3.4. Protein–protein interaction network analysis

**Fig 3:**
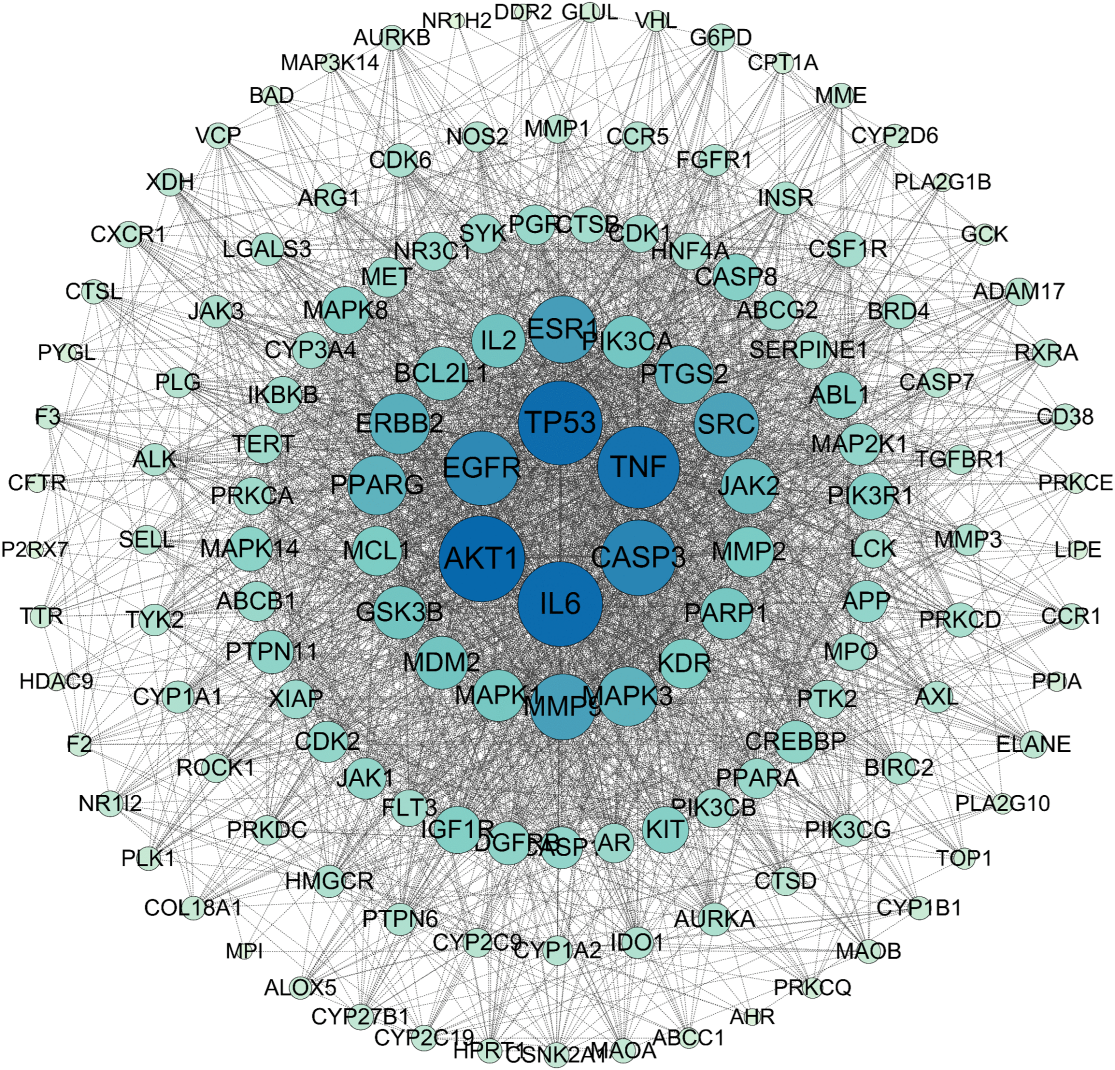
PPI network of intersection targets of *Isatis indigotica* against HBV-associated hepatic inflammation. Node size represents degree centrality; color intensity represents betweenness centrality. The top four hub genes (*AKT1*, *IL6*, *TP53*, *TNF*) are highlighted.

The 142 intersection targets were imported into the STRING database for PPI analysis. The resulting network, optimized in Cytoscape 3.10.3 using the CytoNCA plug-in, comprised 142 nodes and 2,534 edges (Fig 3). Topology analysis demonstrated that nodes with larger degree values occupied more central positions in the network. The top 10 intersection targets ranked by degree value are presented in Table 2; the full ranking is available in S4 Table. The complete filtered PPI interaction dataset is provided in S5 Table. These hub nodes exhibit extremely high connectivity in the network and serve as key signaling nodes, suggesting that *Isatis indigotica* may modulate hepatic inflammatory processes by regulating these hub proteins.

**Table 2:** Top 10 hub targets of *Isatis indigotica* against HBV-associated hepatic inflammation ranked by degree centrality in the PPI network.

Rank	Gene Name	Degree
1	*AKT1*	113
2	*IL6*	111
3	*TP53*	110
4	*TNF*	107
5	*CASP3*	97
6	*EGFR*	95
7	*ESR1*	84
8	*MMP9*	82
9	*SRC*	80
10	*ERBB2*	74

Among the top-ranked targets, *AKT1*, *IL6*, *TP53*, and *TNF* were the four core nodes with the highest degree connectivity in the PPI network. *AKT1*, a member of the serine/threonine kinase family, is a key signaling molecule regulating cell metabolism, survival, proliferation, and protein synthesis. *TNF* and *IL6* are core cytokine genes driving inflammatory cascade reactions. *TP53*, the “guardian of the genome,” not only regulates cell cycle arrest and apoptosis but also participates in inflammatory response regulation by modulating inflammation-related gene expression. The high degree connectivity of these four genes in the PPI network suggests that *Isatis indigotica* may synergistically regulate these hub genes to intervene in downstream inflammatory gene expression programs.

### 3.5. GO and KEGG pathway enrichment analysis

**Fig 4:**
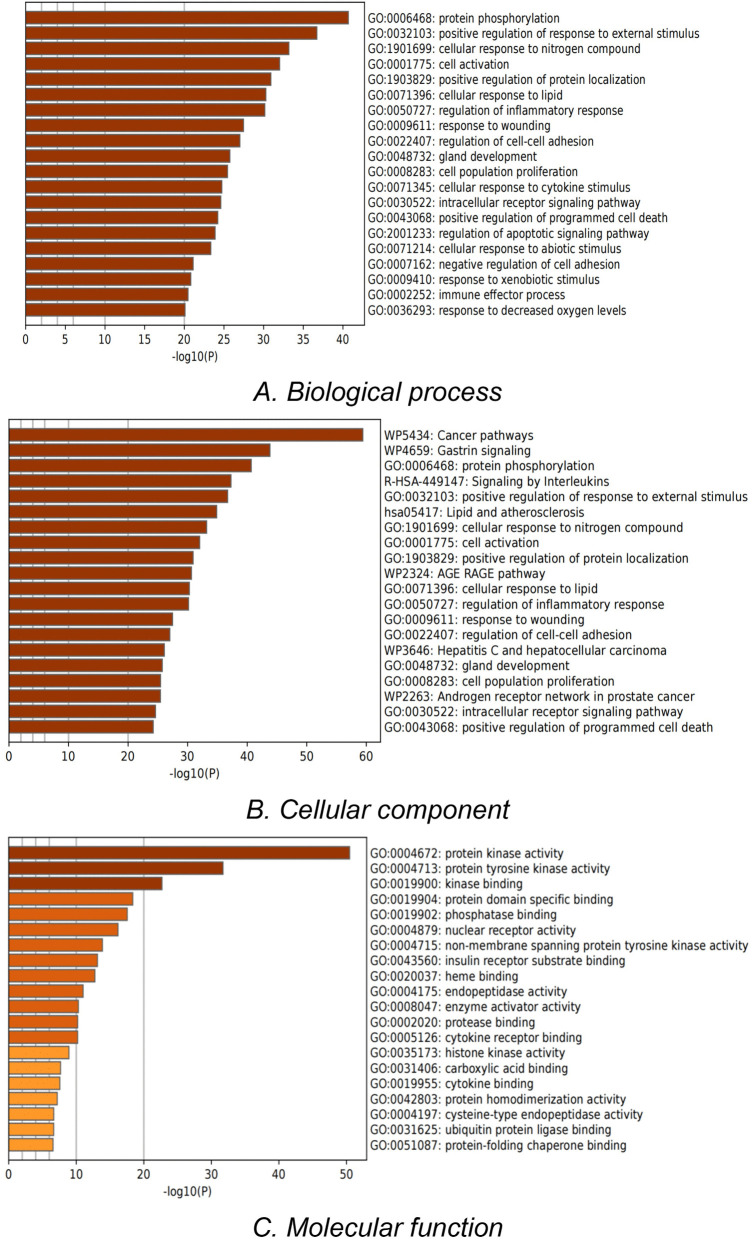
GO enrichment analysis of intersection targets of *Isatis indigotica* against HBV-associated hepatic inflammation. The top 20 terms for biological processes, cellular components, and molecular functions are shown, sorted by ascending P-value. Bar length reflects the number of enriched genes.

GO enrichment analysis of the 142 intersection target genes yielded 5,267 biological process terms, 441 cellular component terms, and 864 molecular function terms. The top 20 most significant terms (sorted by ascending P-value) are shown in Fig 4.

Biological processes were predominantly enriched in protein phosphorylation, positive response to external stimuli, cellular response to nitrogen compounds, cell activation, and positive regulation of protein localization, suggesting that *Isatis indigotica* may modulate the activation status of inflammatory genes through post-translational modifications and cellular stress responses. Molecular functions were significantly enriched in protein kinase activity, protein tyrosine kinase activity, kinase binding, and protein domain-specific binding, indicating that *Isatis indigotica* compounds may regulate inflammatory gene transcription by intervening in kinase-mediated phosphorylation of downstream transcription factors.

**Fig 5:**
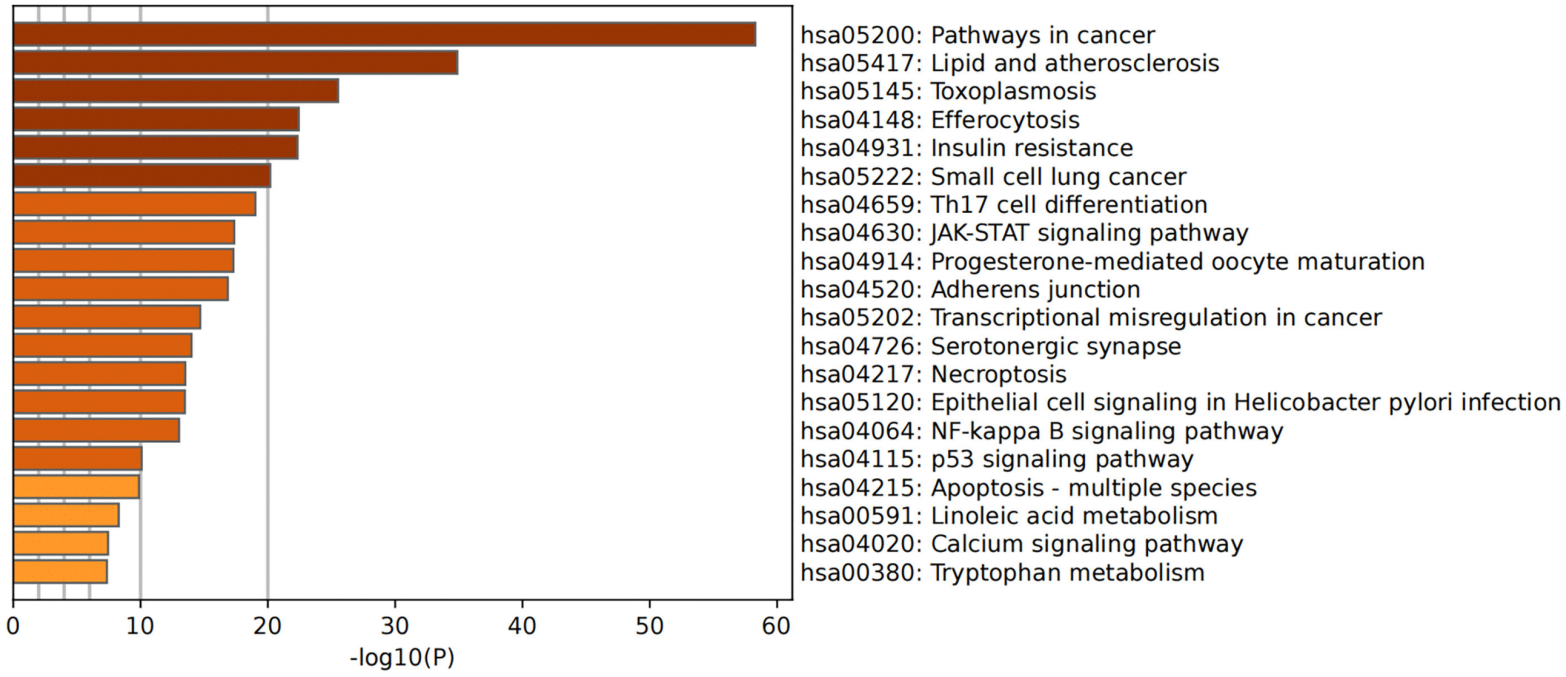
KEGG pathway enrichment analysis of intersection targets of *Isatis indigotica* against HBV-associated hepatic inflammation. The top 20 enriched pathways (*P* < 0.01) are displayed. Color gradient from dark to light represents P-value from low to high (i.e., significance from high to low).

KEGG pathway analysis identified 270 enriched pathways in total. With *P* < 0.01 as the significance threshold, the top 20 pathways are shown in Fig 5. Pathways closely related to the anti-inflammatory mechanism of *Isatis indigotica* include the NF-κB signaling pathway (the core pathway regulating pro-inflammatory gene transcription), the JAK-STAT signaling pathway (the primary cytokine receptor downstream gene activation pathway), the TNF signaling pathway (governing apoptosis and necroptosis), the lipid and atherosclerosis pathway, and the Th17 cell differentiation pathway. Notably, *AKT1*, *IL6*, *TP53*, and *TNF* occupy key node positions in both NF-κB and JAK-STAT pathways. Multi-component simultaneous action on these shared pathway nodes may block inflammatory signal network cascade amplification through a “multi-gene synergistic inhibition” strategy. Complete GO and KEGG enrichment results are provided in S6 Table.

### 3.6. Molecular docking results

**Fig 6:**
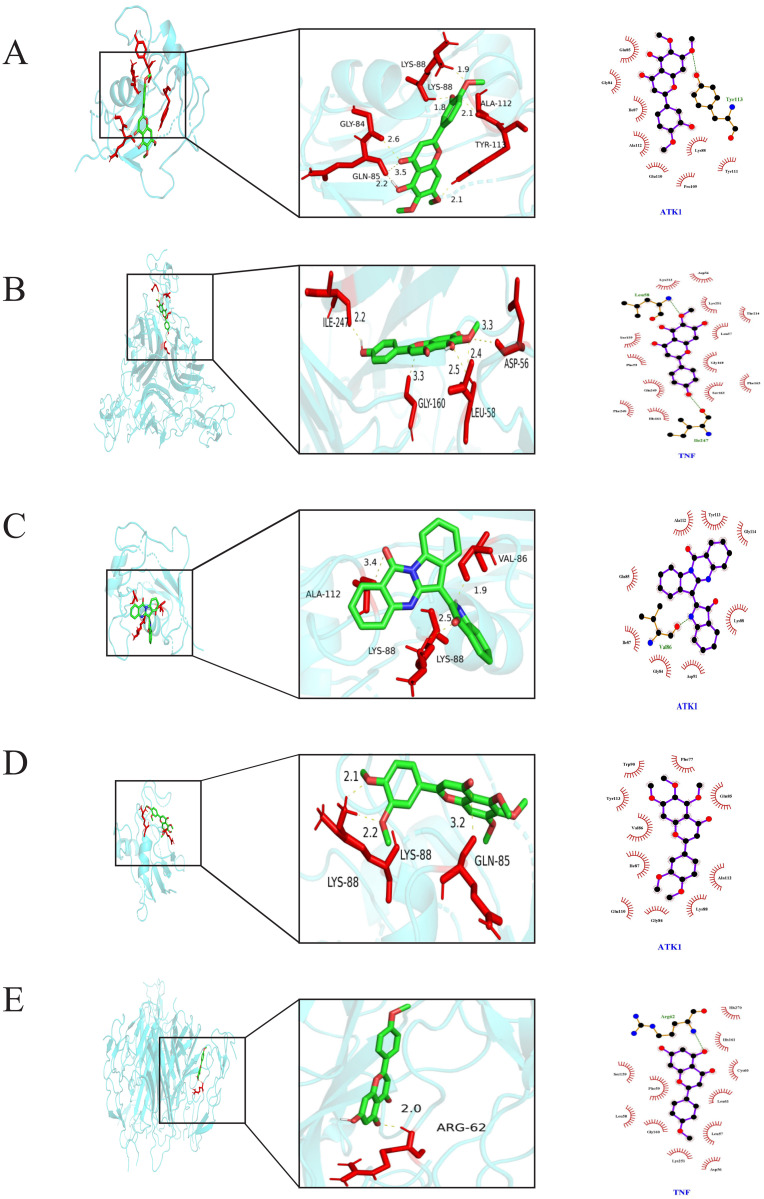
Molecular docking models of core *Isatis indigotica* bioactive compounds with core target proteins. (A) Eupatilin–AKT1 complex showing five binding interactions at GLY-84, GLN-85, LYS-88, ALA-112, and TYR-113; (B) Dinatin–TNF complex showing four interactions at ASP-56, LEU-58, GLY-160, and ILE-247; (C) IQ–AKT1 complex showing three interactions at VAL-86, LYS-88, and ALA-112; (D) Sinensetin–AKT1 complex showing two interactions at GLN-85 and LYS-88; (E) Acacetin–TNF complex showing one interaction at ARG-62.

Molecular docking was performed for the five core bioactive compounds against the four core target proteins (Fig 6). Binding energies of all compound–target pairs were below −5.0 kcal/mol (Table 3), indicating spontaneous formation of stable binding conformations. Among them, IQ showed the lowest binding energy with *AKT1* (−9.35 kcal/mol), suggesting the strongest molecular recognition and binding affinity, which provides a structural basis for its regulatory effect on *AKT1* gene function.

**Table 3:** Molecular docking binding energies (kcal/mol) of core *Isatis indigotica* compounds against core target proteins.

Bioactive compound	AKT1	IL6	TP53	TNF
Acacetin	−6.63	−5.94	−6.0	−7.63
Dinatin	−6.71	−5.14	−5.55	−7.55
Sinensetin	−7.27	−5.13	−5.86	−5.71
Eupatilin	−7.04	−5.29	−6.05	−7.0
IQ	−9.35	−7.35	−8.16	−8.31

### 3.7. Molecular dynamics simulation analysis

Based on molecular docking results, the IQ–AKT1 complex with optimal binding affinity was selected for a 100 ns MD simulation to further validate the long-term stability of the protein–ligand complex.

**Fig 7:**
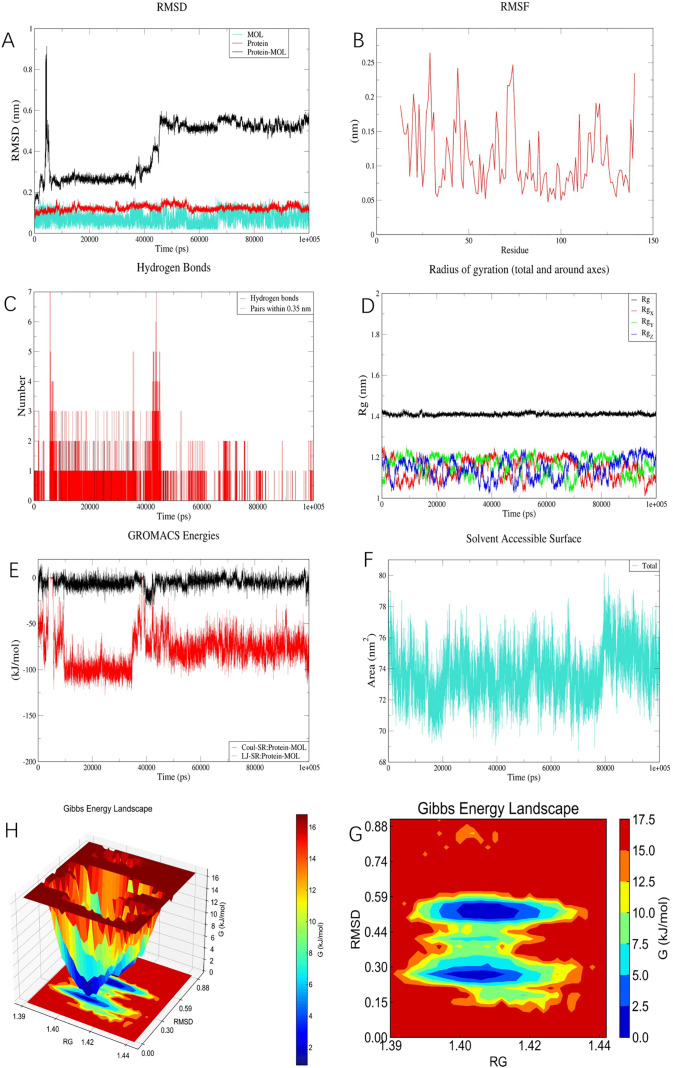
Molecular dynamics simulation analysis of the IQ–AKT1 complex over 100 ns. (A) RMSD of protein backbone (black) and ligand (red); (B) RMSF per residue; (C) number of hydrogen bonds between protein and ligand; (D) Rg of the complex; (E) potential energy contributions (Coulomb and Lennard-Jones); (F) SASA; (G) 2D free energy landscape; (H) 3D free energy landscape. Rg and RMSD were used as reaction coordinates for FEL analysis.

RMSD analysis showed that ligand RMSD fluctuations remained below 0.04 nm throughout the entire 100 ns simulation, indicating overall conformational stability. The protein backbone RMSD converged at approximately 5 ns, and the system entered a stable single trajectory cluster after 40 ns, indicating that thermodynamic equilibrium was reached and that the IQ–AKT1 complex maintained a stable conformation (Fig 7A).

RMSF analysis showed that per-residue atomic displacement fluctuations were predominantly in the range of 0.05–0.2 nm, with overall low protein backbone flexibility and relatively stable side chain movements, favorable for maintaining a stable ligand conformation in the binding pocket (Fig 7B).

Hydrogen bond analysis revealed stable hydrogen bond interactions between the protein and small molecule throughout the simulation, providing key non-covalent binding forces for complex stabilization and supporting the persistence of IQ–AKT1 binding (Fig 7C).

Radius of gyration (Rg) analysis showed that the average Rg of the complex was 1.4 nm, reaching equilibrium rapidly after 5 ns, indicating that the complex maintained good structural compactness and stability with moderate conformational flexibility (Fig 7D).

Potential energy analysis showed that the short-range Coulomb interaction energy was −5.20 kJ/mol and the short-range Lennard-Jones potential energy was −78.27 kJ/mol. Both negative values confirm the presence of strong electrostatic attraction and hydrophobic interactions in the complex, supporting binding stability from an energetic perspective (Fig 7E).

SASA analysis showed that the average SASA of the complex was approximately 74 nm², with only small fluctuations (70–78 nm^2^) throughout the simulation, indicating that the protein–ligand interface remained stable without dissociation or significant conformational changes (Fig 7F).

Free energy landscape (FEL) analysis, using Rg and RMSD as reaction coordinates, showed that the lowest free energy basin was located at Rg = 1.40 nm and RMSD = 0.508 nm (ΔG < 2.5 kcal/mol), presenting a well-defined funnel-shaped potential energy surface. This indicates that the IQ–AKT1 complex preferentially occupies low-energy conformational states during simulation, with multiple interconvertible metastable states, further validating the binding stability of the complex from an energy landscape perspective (Fig 7G and 7H).

## 4. Discussion

This study systematically identified the core bioactive compounds of *Isatis indigotica* against HBV-associated hepatic inflammation using an integrated network pharmacology approach. The five hub compounds—acacetin, dinatin, sinensetin, eupatilin, and IQ—exhibited high degree values in the compound–target network, indicating broad target engagement and polypharmacological potential. Acacetin has been reported to inhibit oxidative stress through the *Keap1*/*Nrf2* pathway and to promote *HSP90*-mediated *COX-2* degradation [16,17], mechanisms that are highly consistent with the well-established ability of flavonoids to enhance hepatic antioxidant defense via the *Nrf2* pathway [18–20]. Dinatin, a 6-methoxyflavone, exhibited a high degree value (48) in the network, suggesting broad target engagement. The polymethoxyflavone eupatilin possesses lipid metabolism-improving and hepatoprotective properties [21]. Our findings suggest that *Isatis indigotica* may exert systemic hepatoprotective effects that potentially involve metabolic pathways beyond the liver; however, as the present study is based solely on computational predictions and lacks metabolomics or systematic omics data, this hypothesis should be interpreted with caution and requires further experimental validation. IQ, an indoloquinazoline alkaloid, showed the strongest binding affinity in molecular docking, representing a novel finding not previously reported in the context of *Isatis indigotica*.

Four core hub targets were identified through PPI network analysis: *AKT1*, *IL6*, *TP53*, and *TNF*. These results align with the study by Deng et al., in which network pharmacology was employed to investigate the effects of *Isatis indigotica* on HBV-associated hepatitis, with common hub targets such as AKT1, IL-6, TP53, and TNF being identified [12]. *AKT1* is an important member of the serine/threonine kinase family, playing key roles in regulating cell metabolism, growth, survival, proliferation, and protein synthesis [22]. In inflammatory responses, AKT signaling regulates downstream effectors including mTOR, eNOS, *FOXO1*, and GSK3β, thereby balancing pro-inflammatory and anti-inflammatory responses and influencing the development of inflammatory diseases [23–26]. Aberrant *AKT1* activation can promote NF-κB nuclear translocation, driving transcriptional upregulation of pro-inflammatory genes including *IL6* and *TNF*. TNF-α is an endogenous cytokine produced by activated monocytes and macrophages [27] that serves as a key mediator of immune and inflammatory responses; serum TNF-α levels are positively correlated with hepatitis disease activity [28,29]. IL-6 is a core regulatory factor in the hepatic inflammation–fibrosis axis, driving inflammatory amplification and fibrotic deposition primarily through the *JAK*/*STAT3* and NF-κB pathways during chronic liver injury [30–32]. *TP53*, as an important tumor suppressor, not only mediates cell cycle arrest and apoptosis [33] but also regulates inflammation-related gene expression [34–37]. In the pathological continuum of “hepatitis–hepatic fibrosis–cirrhosis–hepatocellular carcinoma,” persistent inflammation is the core driver of hepatocyte malignant transformation; *TP53* and *AKT1* together serve as critical gatekeepers at the interface of inflammation and oncogenesis.

The multi-component composition of *Isatis indigotica* may simultaneously act on these four shared hub nodes (*AKT1*, *TNF*, *IL6*, *TP53*), potentially synergistically suppressing the inflammatory gene network through the following mechanisms: (1) interrupting positive feedback amplification by inhibiting TNF-α and IL-6 protein activity; (2) suppressing transcriptional activation by stabilizing *AKT1* structure or inhibiting its kinase activity, thereby reducing phosphorylation-dependent activation of NF-κB and *STAT3* and decreasing transcription levels of downstream pro-inflammatory genes; and (3) restoring cell cycle regulation by facilitating *TP53*-mediated cell cycle arrest or orderly apoptosis of inflammatory-damaged hepatocytes to prevent malignant transformation. Consistently, an independent network pharmacology study on another medicine formula, XingQiChuShiYin (XQCSY), which is used for metabolic dysfunction-associated fatty liver disease (MAFLD) prevention, also identified *AKT1*, *TNF*, *IL-6*, and *TP53* as key hub targets [38].

Beyond direct compound-target interactions, emerging evidence suggests that gut microbiota metabolic reprogramming acts as an early inducer of hepatic inflammatory lesions, and that microbiota-derived metabolites can directly activate hepatocellular inflammatory responses through TLR/NF-κB and JAK/STAT signaling pathways [39]. The hub genes identified in the present study—*AKT1*, *TNF*, and *IL-6*—are precisely the key effector nodes within this microbiota-metabolite-inflammatory signaling axis. This perspective extends the present work from a narrow “compound-target direct interaction” framework toward a broader “microbiota-host co-metabolism” paradigm, suggesting that *Isatis indigotica* may exert its hepatoprotective effects not solely through direct compound-target engagement on hepatocytes, but also through modulation of the gut microbiota-liver metabolic axis. Such a hypothesis provides a rational foundation for future investigations into the anti-inflammatory mechanisms of *Isatis indigotica* from a gut microecological perspective.

GO enrichment analysis further supported the above mechanisms at the gene function level. Significant enrichment of protein phosphorylation and protein kinase activity terms indicates that *Isatis indigotica* primarily intervenes in inflammatory signal transduction at the level of post-translational modifications. Enrichment of cell activation and cytokine-mediated signaling terms reflects regulatory effects on immune cell function-related genes. Significant KEGG enrichment of *JAK-STAT* and *TNF* pathways corroborates the pathological mechanism of abnormal *STAT3* phosphorylation and upregulated pro-inflammatory factor expression following HBV infection [40,41]. Enrichment of the lipid and atherosclerosis pathway suggests crosstalk between hepatic inflammation and lipid metabolism dysregulation, as chronic HBV infection is frequently accompanied by abnormal lipid metabolism that further aggravates hepatic inflammation and fibrotic progression [42].

The IQ–AKT1 complex exhibited the strongest binding affinity in molecular docking (ΔG = −9.35 kcal/mol), and molecular dynamics simulation further validated its binding stability: the protein backbone RMSD converged rapidly, the binding pocket region maintained favorable conformational rigidity, and persistent hydrogen bonds were observed between the protein and ligand throughout the simulation. The free energy landscape displayed a characteristic funnel-shaped potential energy surface, indicating that the complex preferentially occupies low-energy conformational states and that significant dissociation is unlikely. Both the short-range Coulomb energy and Lennard-Jones potential energy were negative, suggesting that electrostatic attraction and hydrophobic embedding jointly constitute the physicochemical basis for the high-affinity IQ–AKT1 binding. IQ may thus stably occupy the *AKT1* active pocket through a dual-mode mechanism involving hydrogen bond anchoring and hydrophobic residue embedding, thereby attenuating the upstream driving effect of *AKT1* on NF-κB and JAK-STAT pathways.

Notably, IQ is a naturally occurring indoloquinazoline alkaloid (tryptanthrin) found in *Isatis indigotica* and Indigo naturalis. Previous proteomics-based studies have demonstrated that tryptanthrin can concurrently regulate TLR4/NF-κB and JAK/STAT3 signaling pathways [43]. Furthermore, Niu et al. observed significant tryptanthrin-mediated suppression of NF-κB/MAPK activation and reduction of TNF-α and IL-6 levels in both in vitro and in vivo pulmonary fibrosis models, findings that align remarkably well with the hub genes and core pathways identified in the present network pharmacology analysis [44]. The present study provides the first structural dynamics evidence at the atomic level for IQ targeting the upstream kinase *AKT1* through molecular docking and MD simulation.

Food and medicine homology components have been shown to act through dual pathways, direct anti-inflammatory activity following gastrointestinal absorption and indirect metabolic regulation via gut microbiota remodeling, which are highly compatible with the oral pharmacokinetic properties of IQ and its multi-target regulatory roles on NF-κB and JAK-STAT cascades [45]. Therefore, future research should not only verify the direct IQ–AKT1 binding and its kinase regulatory mechanism, but also investigate whether the active components of *Isatis indigotica* may indirectly potentiate their systemic anti-inflammatory effects through modulation of the gut microbiota–liver metabolic axis.

Several limitations of this study should be acknowledged. First, the findings are based entirely on computational predictions and require experimental validation, including in vitro cell-based assays and in vivo animal studies, to confirm the proposed mechanisms. Second, the TCMSP screening thresholds (OB ≥ 30%, DL ≥ 0.18), while widely used, may not capture all pharmacologically relevant compounds of *Isatis indigotica*. Third, network pharmacology analyses rely on the completeness and accuracy of publicly available databases, which are subject to ongoing updates. Fourth, the absence of positive reference inhibitors and negative control ligands in the molecular docking experiments limits the quantitative benchmarking of binding specificity; future studies should include appropriate controls to validate the relative binding affinity of IQ to AKT1. Future studies should prioritize experimental validation of the predicted IQ–AKT1 interaction and the multi-target inhibitory mechanism of *Isatis indigotica* active compounds in relevant cell and animal models of HBV-associated hepatic inflammation.

## 5. Conclusion

This study systematically elucidated the bioactive compounds and target gene regulatory mechanisms of *Isatis indigotica* against hepatic inflammation using an integrated network pharmacology, molecular docking, and molecular dynamics simulation approach. The results suggest that *Isatis indigotica* may regulate hub hepatic inflammation genes—including *AKT1*, *TNF*, *IL6*, and *TP53*—through core active components such as acacetin, dinatin, sinensetin, eupatilin, and IQ, by intervening in the NF-κB, JAK-STAT, and TNF inflammatory signaling pathways. Molecular simulation supported stable binding between IQ and AKT1 from a structural dynamics perspective, providing a molecular basis for the gene regulatory functions of these compounds. Given the medicinal and edible homology characteristics of *Isatis indigotica*, future studies can further explore its application as a functional food ingredient for primary prevention in high-risk hepatic inflammation populations. Particularly, IQ, as a candidate active molecule targeting *AKT1*, represents a promising lead compound for the development of anti-hepatic inflammation agents derived from *Isatis indigotica*.

## Supporting information

S1 Table Complete list of 142 intersection targets of *Isatis indigotica* against HBV-associated hepatic inflammation. (XLSX)

S2 Table Top 10 bioactive compounds ranked by degree in the compound-target network. Ranking includes MOL ID, compound name, degree, betweenness centrality, and closeness centrality.
(XLSX)

S3 Table Compound-target network data. Full compound-target interaction dataset used to construct the compound-target network (Fig 2).
(XLSX)

S4 Table Top 10 hub targets ranked by degree centrality in the PPI network. Ranking includes gene symbol, degree, betweenness centrality, and closeness centrality.
(XLSX)

S5 Table Filtered protein-protein interaction data. PPI interactions from the STRING database filtered at combined score ≥ 0.9, used to construct the PPI network (Fig 3).
(TSV)

S6 Table Full GO and KEGG pathway enrichment analysis results for the 142 intersection targets. (XLSX)
